# Exposure to indoor air pollution and adverse pregnancy outcomes in low and middle-income countries: a systematic review and meta-analysis

**DOI:** 10.3389/fpubh.2024.1356830

**Published:** 2024-05-22

**Authors:** Chala Daba, Lakew Asmare, Fekade Demeke Bayou, Mastewal Arefaynie, Anissa Mohammed, Abiyu Abadi Tareke, Awoke Keleb, Natnael Kebede, Yawkal Tsega, Abel Endawkie, Shimels Derso Kebede, Kaleab Mesfin, Eyob Tilahun Abeje, Ermias Bekele Enyew

**Affiliations:** ^1^Department of Environmental Health, College of Medicine and Health Sciences, Wollo University, Dessie, Ethiopia; ^2^Department of Epidemiology and Biostatistics School of Public Health, College of Medicine and Health Science, Wollo University, Dessie, Ethiopia; ^3^Department of Reproductive and Family Health, School of Public Health, College of Medicine and Health Sciences, Wollo University, Dessie, Ethiopia; ^4^Amref Health in Africa, West Gondar Zonal Health Department, Gondar, Ethiopia; ^5^Department of Health Promotion, School of Public Health, College of Medicine and Health Sciences, Wollo University, Dessie, Ethiopia; ^6^Department of Health System and Management, School of Public Health, College of Medicine and Health Sciences, Wollo University, Dessie, Ethiopia; ^7^Department of Health Informatics, School of Public Health, College of Medicine and Health Sciences, Wollo University, Dessie, Ethiopia

**Keywords:** adverse pregnancy outcomes, low birth weight, preterm birth, small for gestational age, stillbirth, biomass fuel

## Abstract

**Introduction:**

Exposure to indoor air pollution such as biomass fuel and particulate matter is a significant cause of adverse pregnancy outcomes. However, there is limited information about the association between indoor air pollution exposure and adverse pregnancy outcomes in low and middle-income countries. Therefore, this meta-analysis aimed to determine the association between indoor air pollution exposure and adverse pregnancy outcomes in low and middle-income countries.

**Methods:**

International electronic databases such as PubMed, Science Direct, Global Health, African Journals Online, HINARI, Semantic Scholar, and Google and Google Scholar were used to search for relevant articles. The study was conducted according to the updated Preferred Reporting Items for Systematic Reviews and Meta-Analysis (PRISMA) guidelines. A random effect model at a 95% confidence interval was used to determine the association between indoor air pollution exposure and adverse pregnancy outcomes using STATA version 14. Funnel plot and Higgs I^2^ statistics were used to determine the publication bias and heterogeneity of the included studies, respectively.

**Results:**

A total of 30 articles with 2,120,228 study participants were included in this meta-analysis. The pooled association between indoor air pollution exposure and at least one adverse pregnancy outcome was 15.5% (95%CI: 12.6–18.5), with significant heterogeneity (I^2^ = 100%; *p* < 0.001). Exposure to indoor air pollution increased the risk of small for gestational age by 23.7% (95%CI: 8.2–39.3) followed by low birth weight (17.7%; 95%CI: 12.9–22.5). Exposure to biomass fuel (OR = 1.16; 95%CI: 1.12–1.2), particulate matter (OR = 1.28; 95%CI: 1.25–1.31), and kerosene (OR = 1.38; 95%CI: 1.09–1.66) were factors associated with developing at least one adverse pregnancy outcomes.

**Conclusions:**

We found that more than one in seven pregnant women exposed to indoor air pollution had at least one adverse pregnancy outcome. Specifically, exposure to particulate matter, biomass fuel, and kerosene were determinant factors for developing at least one adverse pregnancy outcome. Therefore, urgent comprehensive health intervention should be implemented in the area to reduce adverse pregnancy outcomes.

## Introduction

Exposure to indoor air pollution is a persistent public health problem in the 21st century globally. The World Health Organization (WHO) released a report in 2022 indicating that around 3.2 million people die each year as a result of exposure to indoor air pollution. Of this death, more than 237,000 of them were under five children age (1). Exposure to indoor air pollution continues to be the leading cause of adverse pregnancy outcomes such as low birth weight, preterm birth, stillbirth, and neonatal mortality, which could need urgent intervention (2). According to the WHO report of 2019, about 20 million infants were born with low birth weight (LBW) whereas 15 million infants were preterm birth (3).

The magnitudes of adverse pregnancy outcomes are more prevalent in low-income countries because the majority of communities rely on biomass fuel for cooking and heating (4). Evidence from the recent global burden disease released in 2017 indicates that 60.9 million disability-adjusted life years were reported in low and middle-income countries as a result of exposure to air pollution (5). For instance, the odds of stillbirth adverse pregnancy outcomes were 23 times higher among low-income countries than high-income countries (6), which highlights urgent interventions needed. Beyond adverse pregnancy outcomes, exposure to indoor air pollution has a significant effect on cognitive and economic status; which was lost 6.1% of Gross Domestic Product (GDP) in 2022 alone (7).

Various studies have investigated the association between exposure to indoor air pollution and adverse pregnancy outcomes in different parts of the country (4, 8–17). However, the findings from these studies have been inconclusive and varied, which could hinder the implementation of effective intervention strategies to reduce adverse pregnancy outcomes. For instance, studies conducted in Ethiopia (18), Peru (19), and Zambia (20) showed that exposure to indoor air pollution increased the risk of small for gestational age, stillbirth, and miscarriages whereas a non-significant association with neonatal death (18). However, studies conducted in India (13) Indonesia (21) and Bangladesh (22) also showed that there is a significant association between exposure to indoor air pollution and neonatal death and low birth weight.

Moreover, exposure to indoor air pollution and all types of adverse pregnancy outcomes were not pertinently investigated in low and middle-income countries. In addition to this, the previous systematic reviews and meta-analyses on exposure to indoor air pollution did not assess exposures to biomass fuel and particulate matter (PM_10_ and PM_2.5_) together, which could underestimate the magnitude of adverse pregnancy outcomes (23–25). Therefore, the objective of this systematic review and meta-analysis aimed to determine the pooled prevalence of adverse pregnancy outcomes among pregnancy women exposure to indoor air pollution in low and middle-income countries. The results from this meta-analysis will provide essential evidence that can inform adverse pregnancy outcomes control program planners, policymakers, and healthcare providers. This evidence will be valuable in designing and implementing evidence-based interventions aimed at reducing the burden of stillbirth, low birth weight, small gestational age, and preterm birth, neonatal and prenatal mortality.

## Methods and materials

**Registration:** This systematic review and meta-analysis is registered in PRESPERO under the registration number CRD42023432239.

### Study selection, search strategy, and study period

This meta-analysis followed the Preferred Reporting Items for Systematic Reviews and Meta-Analysis Protocols (PRISMA) guidelines (26). To retrieve relevant articles, international electronic databases such as PubMed, Science Direct, Global Health, African Journals Online, HINARI, Semantic Scholar, and Google and Google Scholar searches were used. Gray literature was also identified from different university's digital libraries. The following key terms were used to search the studies: Indoor air pollution”, “household air pollution”, biofuels OR “household fuel” “biomass”, “domestic fuel”, “coal”, “cooking fuel”, “wood”, “cooking smoke”, “charcoal”, “solid fuel”, “cooking firewood”, “biomass fuel”, “biomass smoke”, “wood fuel”, “wood smoke”, “charcoal smoke”, “Air pollution”, “Particulate matter”, “PM_10_”, “PM_2.5_”, “ozone”, “nitrogen dioxide”, “sulfur dioxide”, “carbon monoxide” “Polycyclic Hydrocarbons”, “Pregnancy outcome”, “birth weight”, “low birth weight”, “low birthweight”, “premature birth”, “premature infant”, “fetal growth retardation”, “fetal development”, “gestational age”, “small for gestational age”, “small gestational age”, “fetal mortality”, “fetal death”, “perinatal mortality”, “stillbirth”, “embryo loss”, “spontaneous abortion”, “congenital abnormalities”, “neural tube defects”, “low and middle income country”, “low income country”, “middle income country”, low and middle income countries”. All key terms were combined using the Boolean operators “AND” or “OR” as appropriate. The search was carried out up to December 1, 2023, by four authors independently (CD, YS, AE, and SDK).

### Inclusion and exclusion criteria

In this meta-analysis, we included observational studies (cross-sectional, case-control, and cohort studies) on indoor air pollution exposure and adverse pregnancy outcomes in low and middle-income countries. Studies published between 2000 and December 1, 2023, were included in the meta-analysis. However, qualitative studies, unretrievable studies, editorial letters, studies with poor methodological quality, and studies that did not report the outcome of interest were excluded from the meta-analysis.

### Outcome assessment

The primary outcome of the study was to estimate the pooled association between indoor pollution exposure and adverse pregnancy outcomes in low and middle-income countries, calculated by dividing the number of adverse pregnancy outcomes by the total sample size and multiplying by 100.

### Data extraction and quality assessment

After all searched articles were exported into the Endnote X20 version, and duplicate articles were removed, data was extracted by using a standard data extraction template by four authors (CD, KM, ET, and EB). The standard data extraction template consists of the author's name, country, publication year, exposure assessment, study design, prevalence, type of adverse pregnancy outcome, and sample size. Five reviewers (LA, CD, FD, MA, and AM) screened the relevant articles for eligibility, and the quality of each article was evaluated using the Joana Brigg Institute (JBI) critical appraisal checklist (27). The quality of each study was independently assessed by the four authors (CD, AAT, AK, and NK), with scores measured on a scale of 100%. A quality score of <50% was used to include articles for further analysis (28, 29). In the case of any discrepancies encountered during the quality assessment, the mean score was computed from the evaluations of all reviewers to address and resolve any differences.

### Statistical analysis and synthesis

DerSimonian and Liard's method of random effect model was used to determine the pooled association between indoor air pollution exposure and adverse pregnancy outcomes using STATA 14 (30). The Higgs I^2^ statistic model was used to determine the heterogeneity of the included studies, with values of 25%, 50%, and 75% indicating low, moderate, and high heterogeneity, respectively (31). A *p*-value of less than 0.05 was considered indicative of the presence of heterogeneity. The publication bias was assessed using a funnel plot and Egger's test with a *p*-value less than 0.05 suggesting a publication bias (32).

Subgroup analysis was carried out based on various study characteristics such as sample size (large or small), year of publication (2020 and after or before 2020), quality of the study (high or low quality), and study setting (nationwide, healthcare facility or community level). Moreover, a sensitivity analysis was also performed to assess the influence of a single study on the pooled prevalence estimates.

### Operational definition

**Low birth weight**: a birth weight of <2,500 g (33).

**Stillbirth**: “a baby who dies after 28 weeks of pregnancy but before or during birth”(34).

**Neonatal death**: “deaths among live births during the first 28 completed days of life” (35).

**Preterm birth**: “babies born alive before 37 weeks of pregnancy” (36).

**Small for gestational age**: those smaller in size than normal for their gestational age, most commonly defined as a weight below the 10th percentile for the gestational age (37).

## Results

### Study selection

**Figure 1:**
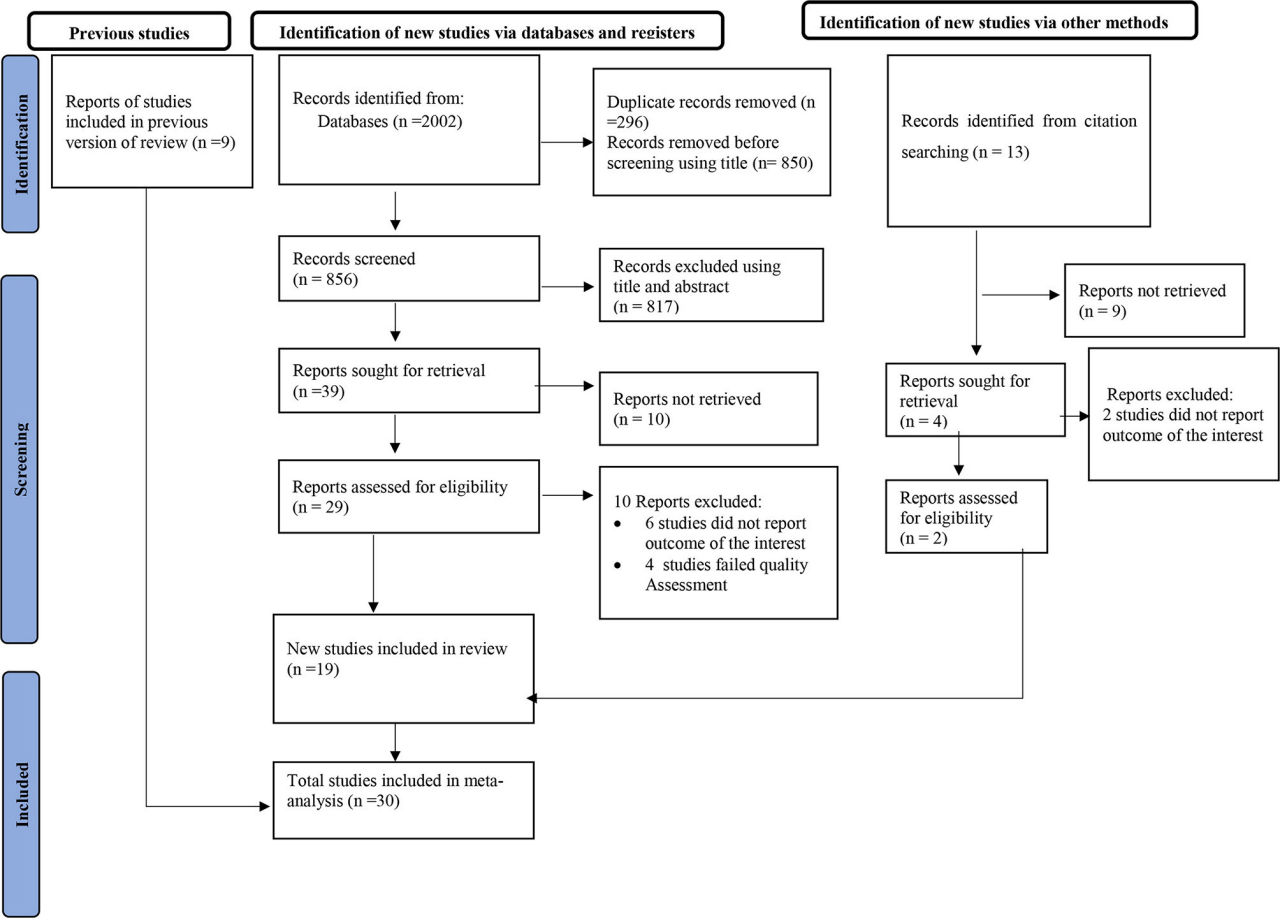
PRISMA flow diagram of the included studies for the systematic review and meta-analysis of exposure to indoor air pollution and adverse pregnancy outcomes in low and middle-income countries, 2023.

Using an international electronic database, a total of 2,002 articles was identified. Out of these, 296 duplicate articles were excluded using the Endnote reference manager and 817 articles were excluded based on their titles and abstracts. Besides, 10 articles were also excluded based on the quality assessment and outcomes of the interest. Finally, 30 full-text articles were eligible for this meta-analysis (Figure 1).

### Characteristics of the included studies

A total of 30 articles (4, 9, 10, 13, 15–17, 20–22, 38–57) were included to determine the association between exposure to indoor air pollution and adverse pregnancy outcomes in low and middle-income countries. In this meta-analysis, a total 2,120,228 of study participants were included. From the included studies, Pakistan had the highest at least one adverse pregnancy outcome (35.4%) (38), and the lowest adverse pregnancy outcome was reported in India (1.7%) (44). Regarding the study setting, 12 studies were conducted in healthcare facilities (4, 13, 16, 21, 38–41, 43, 44, 46, 50), 14 in nationwide (country level) (4, 13, 16, 21, 22, 38–41, 43, 44, 46, 50, 52), and four in community level (17, 47, 51, 54). Likewise, 28 studies had scored 75% and more of JBI the quality assessment (4, 9, 10, 13, 15–17, 20, 22, 38–57), while two studies scored 62.5% (21, 51). Nine studies were carried out in India (10, 13, 16, 44, 47, 52–54, 57), three in China (17, 45, 48), three in Bangladesh (22, 41, 43), three in Ghana (9, 15, 56), two in Pakistan (38, 51), one each in Ethiopia (4), Zimbabwe (46), Nepal (42), Zambia (20), five African countries (India, Pakistan, Guatemala, Kenya, and Zambia) (49), fifteen African countries (39), Nigeria (50), Uganda (40), Indonesia (21), and Sri Lanka (55) (Table 1).

**Table 1:** Descriptive summary of 30 studies included in meta-analysis to estimate the association between exposure to indoor air pollution and adverse pregnancy outcomes in low and middle-income countries, 2023.

**References**	**Country**	**Study setting**	**Exposure assessment**	**Outcome**	**Sample size**	**Prevalence (%)**	**Quality score (%)**
Epuitai et al. (40)	Uganda	Nationwide	Biomass and Kerosene	LBW	15,270	9.6	87.5
Epstein et al. (13)	India	Nationwide	Biomass, coal, kerosene	LBW, neonatal death	36,529	16.5	75
Bachwenkizi et al. (39)	15 Africa country	Nationwide	particulate matter	LBW, PTB	31,594	10	87.5
Ahmed et al. (38)	Pakistan	Nationwide	Biomass fuel	LBW	102,060	35.4	75
Amegah et al. (9)	Ghana	Hospital	Biomass fuel	LBW	647	18.1	87.5
Siddiqui et al. (51)	Pakistan	Community	Biomass fuel	LBW	366	22.7	62.5
Anil K. C. et al. (42)	Nepal	Healthcare facilities	Firewood and Kerosene, LPG, and Bio Gas	LBW	369	NR	87.5
Balakrishnan et al. (10)	India	Healthcare facilities	particulate matter (PM_2.5_)	Birth weight	1,285	16.06	87.5
Li et al. (45)	China	Healthcare facilities	Particulate matter	PTB	1,280,524	8.1	87.5
Sreeramareddy et al. (52)	India	Nationwide	Biomass fuel	LBW	109,041	41	75
Haider et al. (41)	Bangladesh	Nationwide	Biomass fuel	LBW	8,753	17.6	75
Hussein et al. (15)	Ghana	Healthcare facilities	Biomass fuel	LBW, PTB, Neonatal death	1,626	5.5	87.5
Tielsch et al. (54)	India	Community	Biomass fuel and tobacco smoke	LBW, PTB, SGA	11,728	34.07	75
Jiang et al. (17)	China	Community	Biomass fuel	LBW, PTB, SGA	9,895	6.5	87.5
Kanno et al. (4)	Ethiopia	Nationwide	Biomass fuel	LBW	10,014	26.2	87.5
Khan et al. (43)	Bangladesh	Nationwide	Biomass fuel	LBW, Stillbirth, neonatal and infant mortality	22,789	17.7	87.5
Islam et al. (16)	India	Nationwide	Biomass fuel	LBW & birth size	119,537	16.5	87.5
Lakshmi et al. (44)	India	Nationwide	Biomass fuel	Stillbirth	188,917	1.7	87.5
Sunnay et al. (53)	India	Healthcare facilities	NR	LBW	90	NR	75
Mishra et al. (46)	Zimbabwe	Nationwide	Biomass smoke	LBW	6,369	8.46	75
Mukherjee et al. (47)	India	Community	Biomass, Particulate matter	LBW, spontaneous abortion, stillbirth	404	19.6	75
Mulenga et al. (20)	Zambia	Healthcare	Particulate and VOC	LBW, PTB, SGA	1,170	24.8	75
Nisha et al. (22)	Bangladesh	Nationwide	Biomass fuel, agricultural products	Stillbirth and neonatal mortality	27,237	2.8	87.5
Pan et al. (48)	China	Healthcare	Biomass fuel	LBW, PTB, Stillbirth	9,505	5.5	87.5
Patel et al. (49)	India, Pakistan, Guatemala, Kenya, Zambia	Healthcare	Cooking fuel	Stillbirth, prenatal and neonatal mortality	65,912	2.6	87.5
Roberman et al. (50)	Nigeria	Nationwide	Biomass/unclean cooking fuel	LBW, stillbirth and PTB	41,821	14.9	87.5
Suryadhi et al. (21)	Indonesia	Nationwide	Biomass, kerosene, biogas, electricity	LBW, neonatal, and infant death	14,475	6.6	62.5
Vakalopoulos et al. (55)	Sri Lanka	Healthcare	Biomass fuel	LBW, SGA	385	13	87.5
Weber et al. (56)	Ghana	Healthcare	Biomass fuel, Liquid Petroleum Gas, Kerosene	LBW, SGA, PTB, perinatal mortality	772	11.1	87.5
Wylie et al. (57)	India	Healthcare	Biomass fuel	LBW, stillbirth, SGA and PTB	1,199	23.9	75

NR, not reported; PTB, preterm birth; LBW, low birth weight; SGA, small for gestational age.

### Association between indoor air pollution exposure and adverse pregnancy outcomes

**Figure 2:**
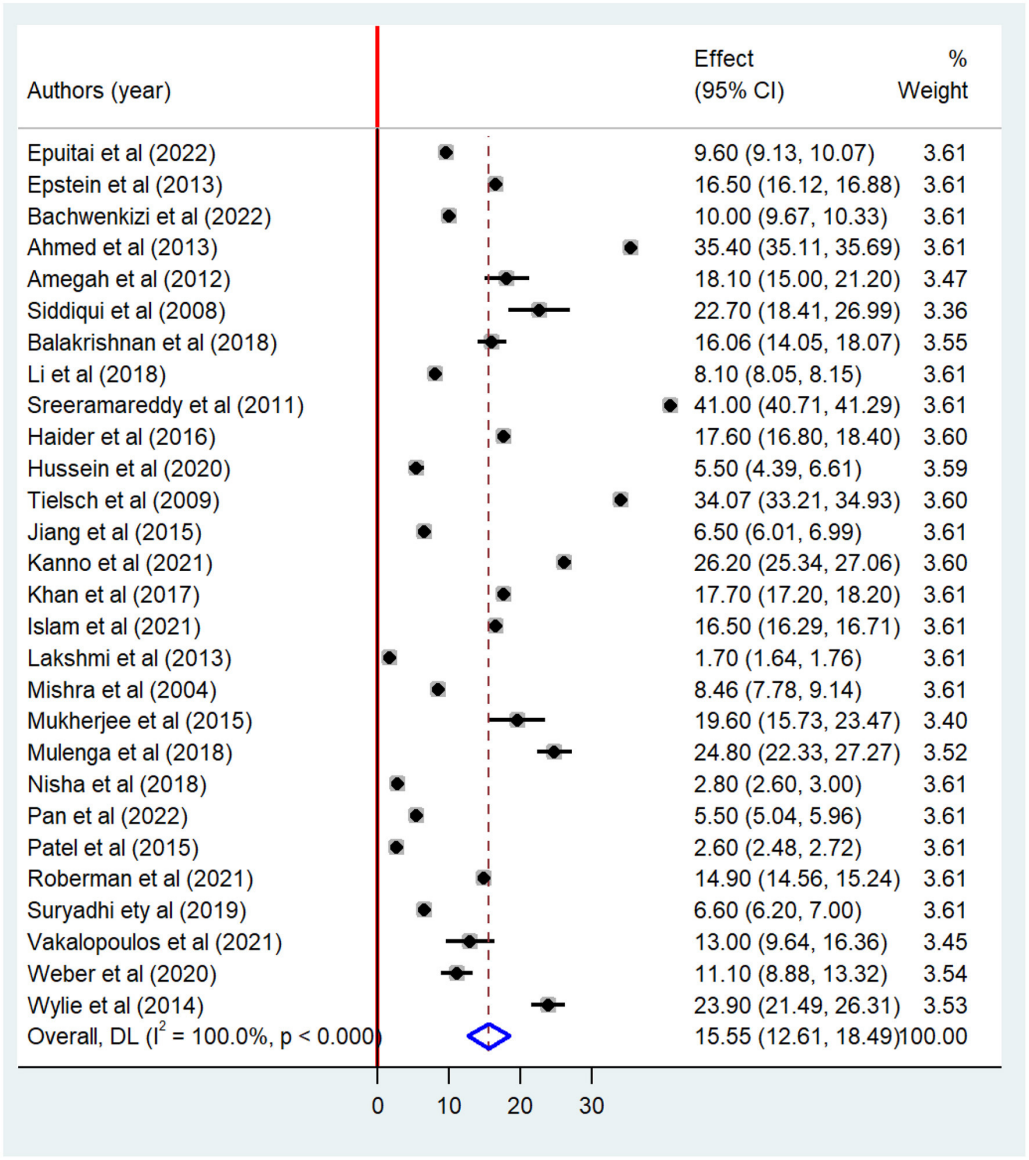
Forest plot of the pooled association between indoor air pollution exposure and adverse pregnancy outcomes in low and middle-income countries, 2023.

Our analysis of the 30 included studies revealed significant heterogeneity among them (I^2^ = 100%; *p* < 0.001). Hence, DerSimonian and Liard's method of random effect model was carried out to estimate the pooled association between exposure to indoor air pollution and adverse pregnancy outcomes. The results of the random effect model indicated the pooled prevalence of at least one adverse pregnancy outcome was 15.55% (95% CI: 12.61–18.49) (Figure 2).

Meta-analysis showed that there is a significant association between indoor air pollution exposure and adverse pregnancy outcomes. The high pooled prevalence of adverse pregnancy outcomes was small for gestational age (23.77%) followed by low birth weight (17.74%). The pooled prevalence of preterm birth among pregnant women exposed to indoor air pollution was 16.56%. Likewise, there is a significant association between indoor air pollution exposure and stillbirth (6.11%) (Table 2).

**Table 2:** Pooled adverse pregnancy outcomes among pregnancy women exposed to indoor air pollution in low and middle-income countries, 2023.

**Type of adverse effect**	**Number of studies**	**Pooled adverse pregnancy outcome (95% CI)**	**Heterogeneity**	
			**I** ^2^	***p*** **-value**
Low birth weight	25	17.74 (12.97–22.52)	100%	< 0.001
Small for gestational age	7	23.77 (8.25–39.30)	99.9%	< 0.000
Preterm birth	9	16.56 (11.51–21.60)	99.9%	< 0.000
Neonatal death	6	2.48 (1.37–3.60)	99.6%	< 0.000
Stillbirth	7	6.11 (3.58–8.65)	99.9%	< 0.000

### Publication bias assessment

**Figure 3:**
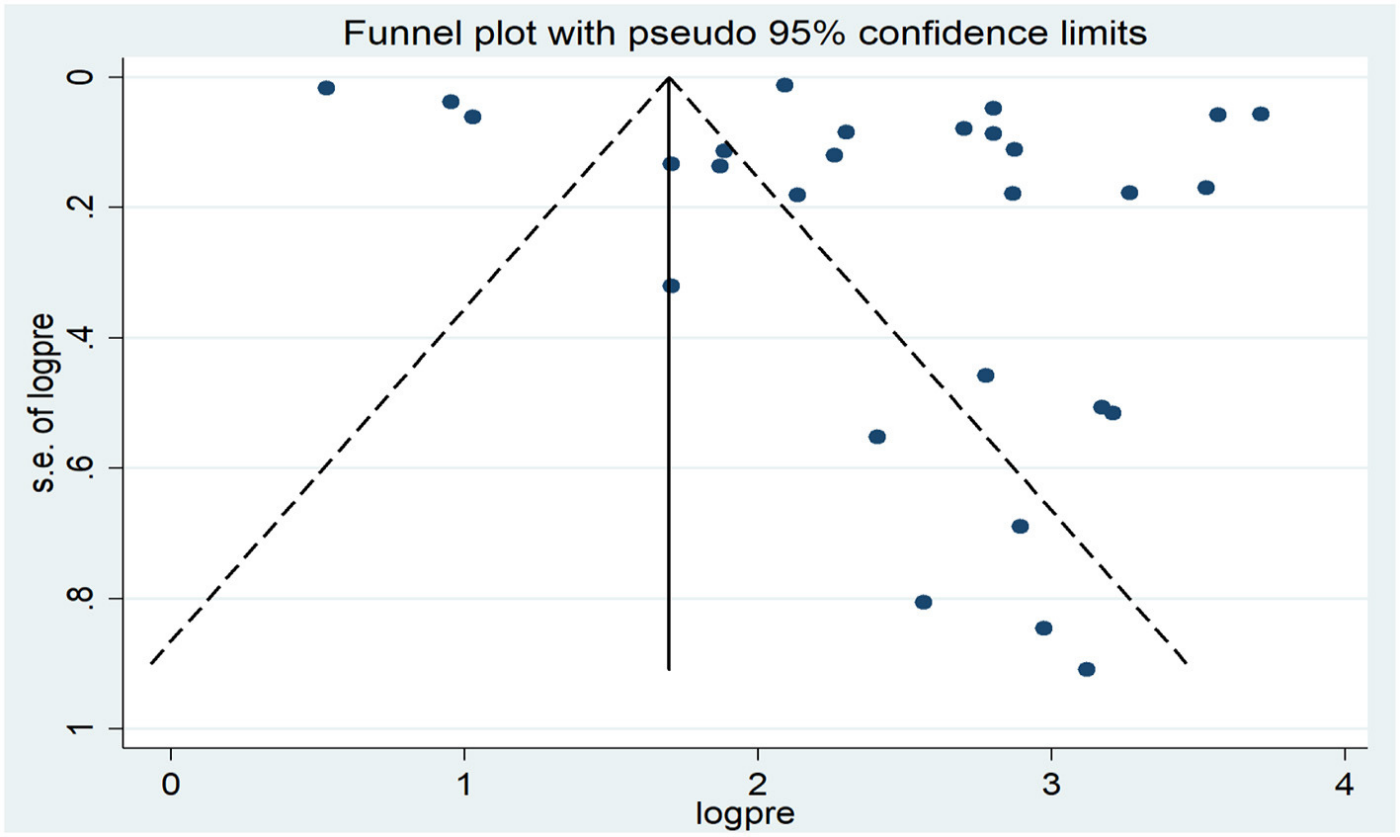
Funnel plot of the pooled prevalence of at least one adverse pregnancy outcomes in low and middle-income countries, 2023.

**Figure 4:**
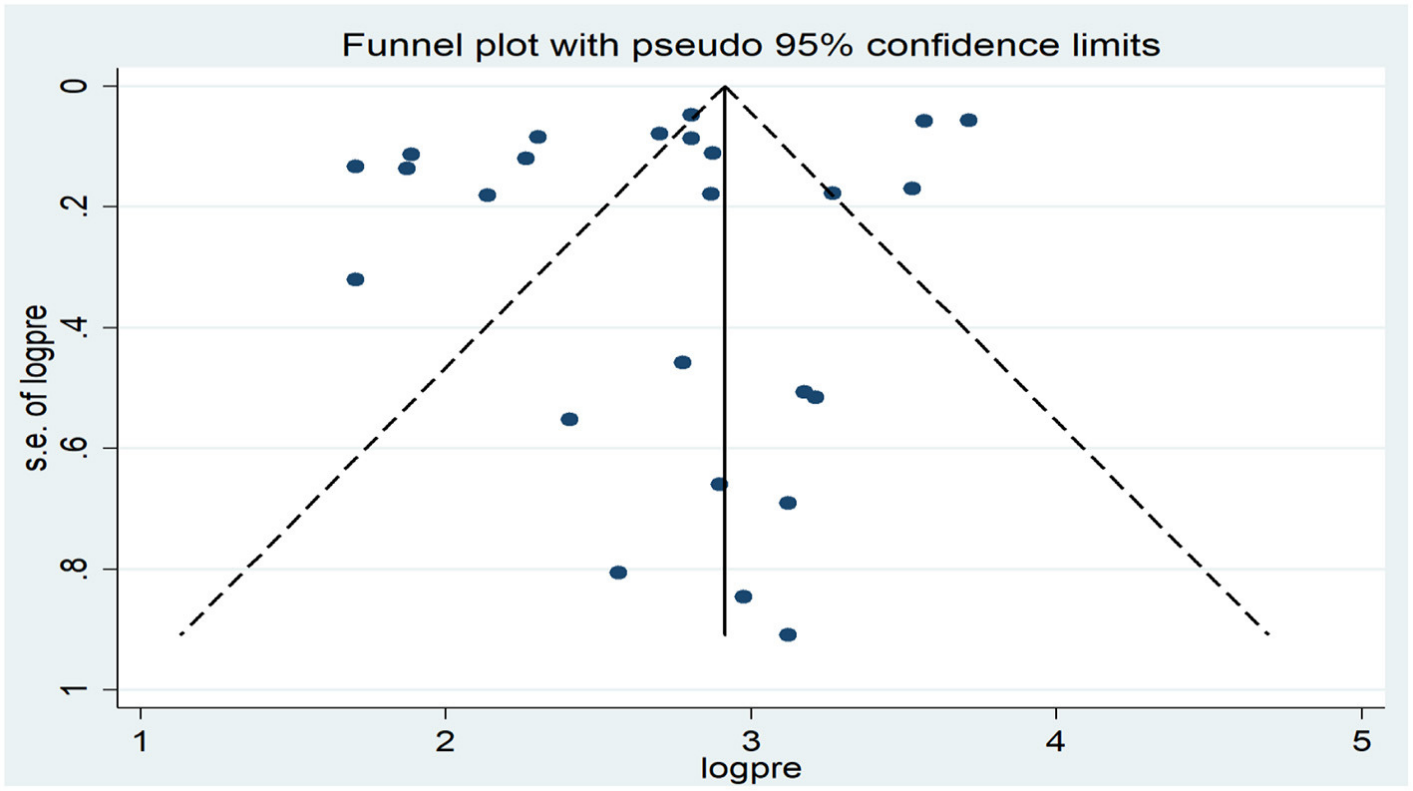
Funnel plot of the pooled association between indoor air pollution exposure and low birth weight in low and middle-income countries, 2023.

A funnel plot was used to determine the publication bias, revealing an asymmetric distribution that strongly indicated the presence of publication bias (Figure 3). Further, statistical analysis employing the Egger regression test verified the absence of publication bias (*p* = 0.159). Similarly, the publication bias was also carried out using a funnel plot and statistical Egger test for low birth weight. The finding showed there was an asymmetric distribution that strongly indicated the presence of publication bias (Figure 4). However, the Egger regression test confirmed the absence of publication bias (*p* = 0.191).

Besides, the publication bias was also determined for small gestational age, preterm birth, and neonatal death using a funnel plot (Supplementary material 1). The Egger regression test confirmed the absence of publication bias among included studies for small gestational age (*p* = 0929) and preterm birth (*p* = 0.891), neonatal death (*p* = 0.322).

### Subgroup analysis

To pinpoint the source of heterogeneity among included studies (I^2^ = 100%, *p* < 0.001), subgroup analysis was conducted based on study setting (nationwide, healthcare facility or community), sample size (small or large), quality of the study (high or low) and year of publication (2020 and after or before 2020). The study conducted at the community level had higher pooled adverse pregnancy outcomes (20.71%), with extreme heterogeneity among included studies (I^2^ = 99.9%, *p* < 001) followed nationwide (16.06%) (Table 3). Regarding the year of publication, the highest pooled prevalence of adverse pregnancy outcomes was observed among studies conducted before 2020 (17.01%; 95%CI: 13.39–29.63) than studies conducted after 2020 (12.48%; 95% CI: 9.15–15.80). In addition, the high pooled prevalence of adverse pregnancy outcomes was observed among small sample size studies (16.71%; 95%CI: 12.46–20.96) as compared to studies conducted with large sample size (15.28%; 95%CI: 12.04–18.52) (Table 3).

**Table 3:** Subgroup analysis of the pooled prevalence of adverse pregnancy outcomes among pregnant women exposed to indoor air pollution in low and middle-income countries, 2023.

**Variables**	**Number of studies**	**OR (95% CI)**	**Heterogeneity**	
			**I^2^**	***p*-value**
**Study setting**				
Nationwide	14	16.06 (9.0–23.13)	100%	< 0.001
Healthcare facilities	10	12.51 (10.09–15.04)	99.9%	< 0.001
Communities	4	20.71 (2.32–39.11)	99.9%	< 0.001
**Sample size**				
Small	5	16.71 (12.46–20.96)	88.5%	< 0.001
Large	23	15.28 (12.04–18.52)	100%	< 0.001
**Quality of the study**				
High	17	10.86 (8.57–13.51)	100%	< 0.001
Low	11	22.79 (13.96–31.62)	100%	< 0.001
**Year of publication**				
2020 and after	9	12.48 (9.15–15.80)	99.8%	< 0.001
Before 2020	19	17.01 (13.39–29.63)	100%	< 0.001

### Sensitivity analysis

**Figure 5:**
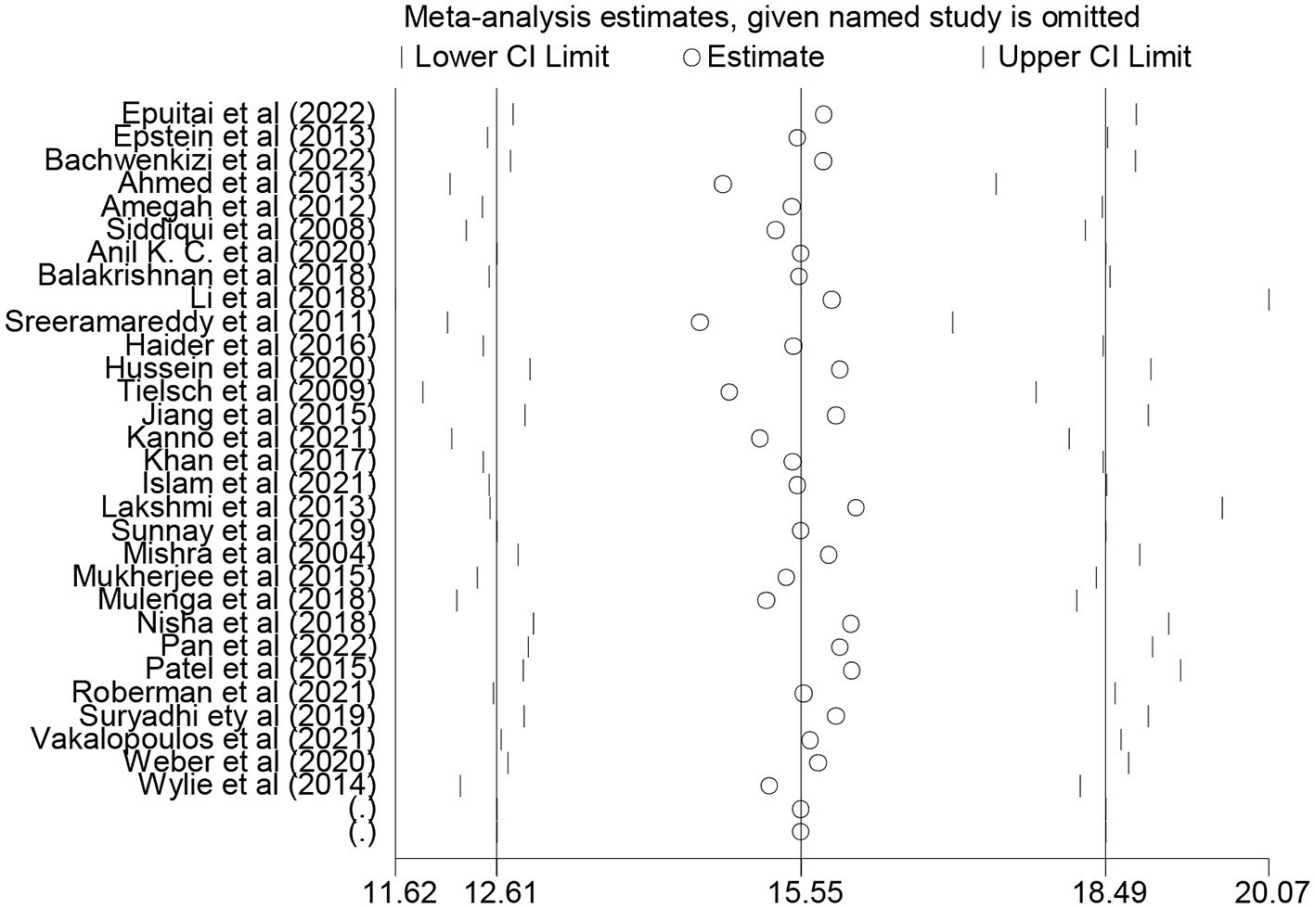
Sensitivity analysis of the pooled association between indoor air pollution exposure and adverse pregnancy outcomes in low and middle-income countries, 2023.

A sensitivity analysis was performed to evaluate the impact of individual studies on the overall pooled estimate of adverse pregnancy outcomes, and the results indicated that no single study exerted a significant effect (Figure 5). Similarly, we also evaluate the impact of individual studies on the overall pooled estimate of low birth weight, small for gestational age, preterm birth, neonatal death, and stillbirth, and the findings suggest that there is no evidence of a single study's effect on the overall pooled prevalence (Supplementary material 2).

### Factors associated with adverse pregnancy outcomes

**Figure 6:**
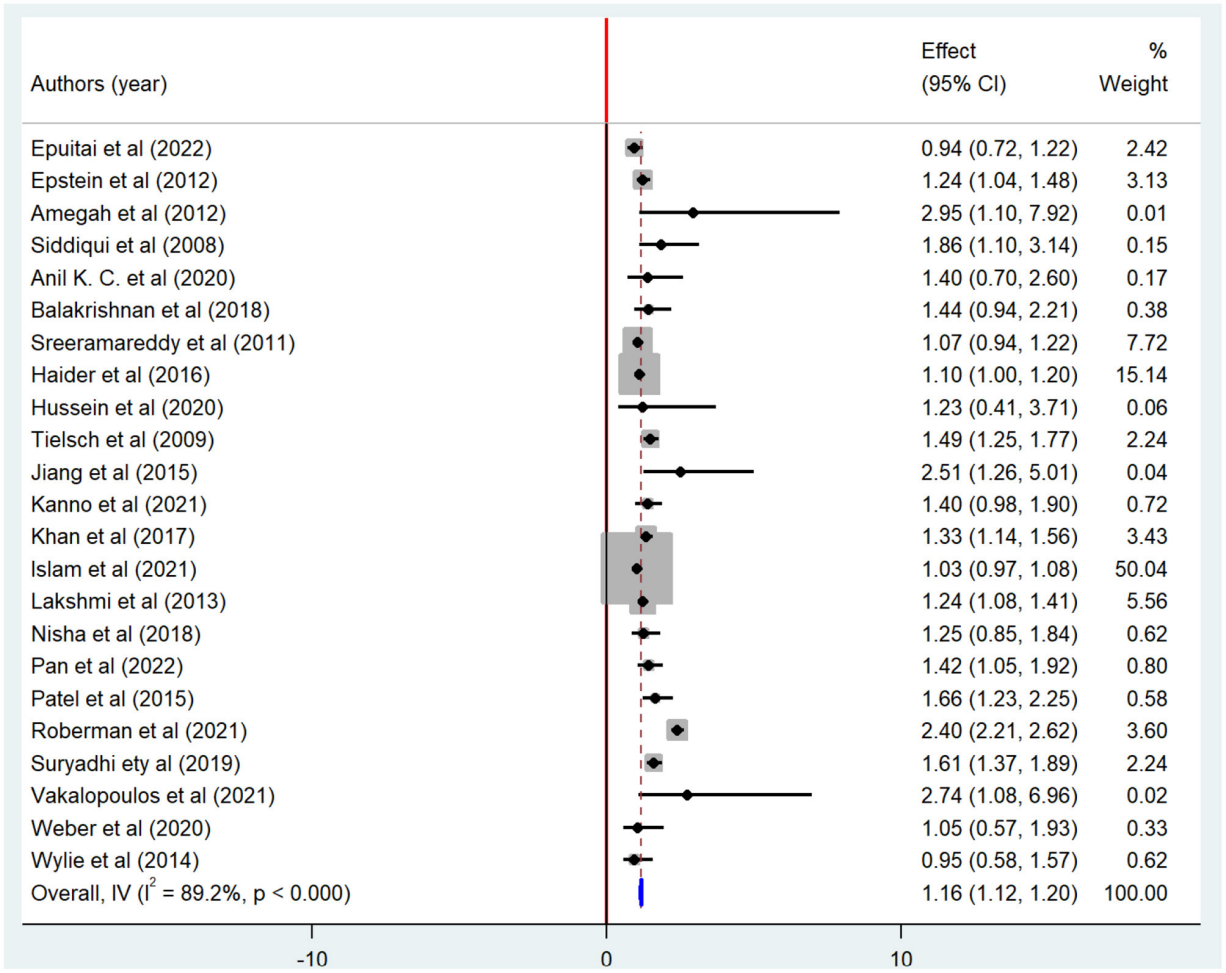
Forest plot of odds ratio for the association between biomass fuel exposure and adverse pregnancy outcome, 2023.

Exposure to indoor air pollution, such as biomass fuel, particulate matter, and kerosene was statistically significantly associated with adverse pregnancy outcomes. Twenty-three studies were included to determine the association between biomass fuel exposure and adverse pregnancy outcomes (4, 9, 10, 13, 15–17, 21, 22, 40–44, 48–52, 54–57). Thirteen of the included studies had a positive association (9, 13, 17, 21, 41, 43, 44, 48–51, 54, 55), while negative association in 10 studies. The pooled results from random effect analysis showed that exposure to biomass fuel would increase the risk of adverse pregnancy outcomes by 1.16 (OR = 1.16; 95% CI: 1.12–1.20), with significant heterogeneity (I^2^ = 88.8%; *p* < 0.001) (Figure 6).

The association between particulate matter exposure and adverse pregnancy outcomes was determined based on four studies (20, 39, 47, 56). Two of the included studies had a positive association (39, 47), while negative association in the remaining two studies (20, 56). The odds of adverse pregnancy outcomes were 1.28 times higher among pregnant women who had particulate matter exposure than those who had no exposure (OR = 1.28; 95% CI: 1.25–1.31), with significant heterogeneity (I^2^= 70.1%; *p* = 0.018) (Table 4).

**Table 4:** The pooled factors associated with at least one adverse pregnancy outcome in low and middle-income countries, 2023.

**Variables**	**Number of studies**	**OR (95% CI)**	**Heterogeneity**	
			**I** ^2^	***P*** **-value**
Biomass fuel	23	1.16 (1.12–1.20)	88.8%	< 0.001
Particulate matter	4	1.28 (1.25–1.31)	70.1%	0.018
Kerosene	2	1.38 (1.09–1.66)	0.0%	< 0.000

Similarly, the association between kerosene exposure and adverse pregnancy outcomes was determined based on the finding of two studies (13, 44). There is a significant association in one study (44) and a non-significant in another study (13). Meta-analysis showed that the pregnant women who had kerosene exposure were 37% times more likely to have adverse pregnancy outcomes than counterparts (OR = 1.37; 95%CI: 1.09–1.66) (Table 4).

## Discussion

This systematic review and meta-analysis aimed to determine the association between indoor air pollution exposure and adverse pregnancy outcomes in low and middle-income countries. The overall pooled prevalence of at least one adverse pregnancy outcome was found to be 15.5% (95% CI: 12.61–18.49); with extreme heterogeneity among included studies (I^2^ = 100; *p* < 0.001). Specifically, exposure to indoor air pollution increased the risk of small gestational age by 23.7% (95%CI: 8.25–39.3), stillbirth (6.11%; 95%CI: 3.58–8.65), and neonatal mortality (2.48%; 95%CI: 1.37–3.60). This finding is corroborated by recent systematic reviews and meta-analyses (5, 58, 59). This is mainly due to exposure to carbon monoxide, particulate matter, and highly polluting biomass fuel could enhance the burden of adverse pregnancy outcomes (55).

There seems to be evidence that exposure to indoor air pollution has a significant contribution to stillbirth outcomes (2), which is consistent with the current meta-analysis. Similarly, our finding was supported by a recent study conducted in Ethiopia (18), which indicates that there is an association between exposure to indoor pollution and stillbirth. This might be due to the pregnant women being exposed to high-polluting indoor air pollution, which could contribute to a significant association with adverse pregnancy outcomes (stillbirth) (18).

The current meta-analysis showed that one in six neonates could experience low birth weight (17.7%; 95%CI: 12.97–22.52), which is consistent with previous study conducted in Sub-Saharan Africa (14%) (60). The current finding was also supported by the previous meta-regression analysis of 204 countries (25), which indicates that exposure to ambient and household indoor air pollution PM_2.5_ increased the risk of all low birth weight by 15.6%. This is mainly due to exposure to high polluting air pollution especially, anthropogenic particulates (PM_2.5_) harmful to child health and growth, leading to low birth weight.

The odds of at least one adverse pregnancy outcome were 1.16 times higher among women who used high-polluting biomass fuel than those who used non-polluting biomass. Pregnant women exposed to highly polluting biomass fuels (namely, firewood and kerosene) have statistically significant factors for infants with low birth weight showing that low birth weight infants were sixteen percent times as likely to be born mothers exposed to high polluting biomass fuel than counterparts; which is consistent with the recent studies conducted by Kadam et al. (61). Increased exposure time to indoor air pollutants such as particulate matter, carbon monoxide, and sulfur dioxide has a significant impact on the growth and development of the fetus and subsequently low birth weight (62). The association between exposure to indoor air pollution and low birth weight is also established study done in developing countries (62). Therefore, mitigation measures such as cooking outdoors, decreasing exposure time, and improving the ventilation system with chimneys might play a significant role in decreasing adverse pregnancy outcomes (63).

The pooled prevalence of preterm birth was found to be 16.56%; these high pregnancy outcomes might be due to the exposure to highly polluting biomass fuel than non-polluting fuel; which is supported by a study conducted in East India (57). The odds of at least adverse pregnancy outcomes were 1.28 times higher among pregnant women exposed to particulate matter than those not exposed to particulate matter. The current finding was also consistent with a study conducted in Ohio, which indicates that pregnancy women exposure to high particulate matter (PM_2.5_) could increase the risk of preterm by 1.19 (64). A recent meta-analysis also confirmed that exposure to particulate matter (PM_2.5_) could increase the risk of preterm birth by 1.10 (65). This is mainly because cooking with highly polluting biomass fuel in poorly ventilated homes generates high concentrations of particulate matter (10). Hence, it is important to reduce exposure time to household air pollution may be crucial for the reduction of adverse pregnancy outcomes.

In this meta-analysis, we found that there is a significant association between exposure to indoor air pollution and neonatal mortality. The pooled prevalence of neonatal mortality was found to be 2.48%, with extreme heterogeneity (I^2^ = 99.9%; *p* < 0.001). This finding was in line with studies conducted in Bangladesh (43) and Pakistan (66). This finding was also supported by a study conducted in five world regions (67), which indicates that exposure to indoor air pollution increased the risk of neonatal mortality by 1.24 (95% CI: 1.14–1.34). This is mainly due to exposure to air pollution, which entails potential hazards for their neonates like lower birth weight, preterm birth, and lung developmental defects causing onset of respiratory diseases and reduced lung function in children (68–70). Because neonates need more air intake for survival and therefore inhale excessive oxygen as compared to children; air polluted with unwanted contaminants enters their lungs and provokes consequent deaths (70). It is also well documented that exposure to air pollution has a significant and positive association with neonatal mortality, particularly in low and middle-income countries (71).

### Limitations and strengths of the study

The study followed the updated preferred reporting items for systematic review and meta-analysis. In this meta-analysis, all types of adverse pregnancy outcomes as a result of exposure to indoor air pollution were pertinently assessed. One of the limitations of this meta-analysis was that it did not establish causality between independent and dependent variables since the majority of the included studies were cross-sectional and case-control study designs.

## Conclusions

We found that there was a significant association between indoor air pollution exposure and adverse pregnancy outcomes. The pooled adverse pregnancy outcomes among women exposed to indoor air pollution were found to be high; which calls for urgent interventions, particularly in low and middle-income countries, where cooking with biomass fuels is common. Therefore, the Ministry of Health, healthcare workers, and other concerned bodies should provide comprehensive public health intervention to reduce adverse pregnancy outcomes. Besides, mechanistic studies are needed to understand the underlying mechanisms of association between exposure to indoor air pollution and adverse pregnancy outcomes. Further studies on the toxicological effect on indoor air pollution (particulate matter, biomass fuel, kerosene) are needed to verify these findings.

## Data availability statement

The original contributions presented in the study are included in the article/Supplementary material, further inquiries can be directed to the corresponding author.

## Author contributions

CD: Conceptualization, Data curation, Formal analysis, Funding acquisition, Investigation, Methodology, Project administration, Resources, Software, Supervision, Validation, Visualization, Writing—original draft, Writing—review & editing. LA: Conceptualization, Investigation, Methodology, Supervision, Validation, Visualization, Writing—review & editing. FD: Conceptualization, Investigation, Resources, Software, Supervision, Validation, Writing—review & editing. MA: Conceptualization, Data curation, Formal analysis, Investigation, Supervision, Validation, Visualization, Writing—review & editing. AM: Data curation, Formal analysis, Funding acquisition, Investigation, Methodology, Supervision, Validation, Visualization, Writing—review & editing. AT: Investigation, Methodology, Project administration, Resources, Supervision, Validation, Visualization, Writing—review & editing. AK: Data curation, Formal analysis, Investigation, Resources, Software, Supervision, Validation, Writing—review & editing. NK: Conceptualization, Data curation, Methodology, Resources, Supervision, Validation, Visualization, Writing—review & editing. YT: Data curation, Funding acquisition, Investigation, Methodology, Resources, Software, Visualization, Writing—review & editing. AE: Data curation, Investigation, Methodology, Resources, Software, Supervision, Validation, Visualization, Writing—review & editing. SK: Conceptualization, Data curation, Investigation, Methodology, Project administration, Resources, Validation, Visualization, Writing—review & editing. KM: Conceptualization, Funding acquisition, Investigation, Methodology, Project administration, Validation, Visualization, Writing—review & editing. EA: Data curation, Formal Analysis, Investigation, Methodology, Project administration, Software, Supervision, Validation, Writing—review & editing. EB: Conceptualization, Formal Analysis, Investigation, Methodology, Software, Validation, Visualization, Writing—review & editing.

## References

[1] WHO. World Health Organization: Fact Sheet: Household Air Pollution. (2022). Available online at: https://www.who.int/news-room/fact-sheets/detail/household-air-pollution-and-health

[2] Amegah AK Quansah R Jaakkola JJ. Household air pollution from solid fuel use and risk of adverse pregnancy outcomes: a systematic review and meta-analysis of the empirical evidence. PLoS ONE. (2014) 9:e113920. 10.1371/journal.pone.0113920 25463771 PMC4252082

[3] WHO. World Health Organization Recommendations for Care of the Preterm or Low Birth Weight Infant. Geneva: World Health Organization. (2022). 36449655

[4] Kanno GG Anbesse AT Shaka MF Legesse MT Andarge SD. Effect of biomass fuel use and kitchen location on maternal report of birth size: cross-sectional analysis of 2016 Ethiopian Demographic Health Survey data. Public Health Pract (Oxf). (2021) 2:100211. 10.1016/j.puhip.2021.100211 36101582 PMC9461598

[5] Lee KK Bing R Kiang J Bashir S Spath N Stelzle D . Adverse health effects associated with household air pollution: a systematic review, meta-analysis, and burden estimation study. Lancet Global Health. (2020) 8:e1427–e34. 10.1016/S2214-109X(20)30343-0 33069303 PMC7564377

[6] Kiguli J Munabi IG Ssegujja E Nabaliisa J Kabonesa C Kiguli S . Stillbirths in sub-Saharan Africa: unspoken grief. Lancet. (2016) 387:e16–e8. 10.1016/S0140-6736(15)01171-X 26794074

[7] Bank W. The Global Health Cost of PM_2.5_ Air Pollution: A Case for Action Beyond 2021. Washington, DC: The World Bank. (2022).

[8] Abusalah A Gavana M Haidich A-B Smyrnakis E Papadakis N Papanikolaou A . Low birth weight and prenatal exposure to indoor pollution from tobacco smoke and wood fuel smoke: a matched case–control study in Gaza strip. Matern Child Health J. (2012) 16:1718–27. 10.1007/s10995-011-0851-4 21842400

[9] Amegah AK Jaakkola JJ Quansah R Norgbe GK Dzodzomenyo M. Cooking fuel choices and garbage burning practices as determinants of birth weight: a cross-sectional study in Accra, Ghana. Environmental Health. (2012) 11:1–10. 10.1186/1476-069X-11-78 23075225 PMC3533864

[10] Balakrishnan K Ghosh S Ganguli B Sambandam S Bruce N Barnes DF . State and national household concentrations of PM_2.5_ from solid cookfuel use: results from measurements and modeling in India for estimation of the global burden of disease. Environm Health. (2013) 12:1–14. 10.1186/1476-069X-12-77 24020494 PMC3851863

[11] Chaudhary N Yadav SN Kalra SK Pathak S Gupta BK Shrestha S . Prognostic factors associated with small for gestational age babies in a tertiary care hospital of Western Nepal: a cross-sectional study. Health Sci Rep. (2021) 4:e250. 10.1002/hsr2.250 33614985 PMC7883381

[12] Demelash H Motbainor A Nigatu D Gashaw K Melese A. Risk factors for low birth weight in Bale zone hospitals, South-East Ethiopia: a case–control study. BMC Pregn Childbirth. (2015) 15:1–10. 10.1186/s12884-015-0677-y 26463177 PMC4604703

[13] Epstein MB Bates MN Arora NK Balakrishnan K Jack DW Smith KR. Household fuels, low birth weight, and neonatal death in India: the separate impacts of biomass, kerosene, and coal. Int J Hyg Environ Health. (2013) 216:523–32. 10.1016/j.ijheh.2012.12.006 23347967

[14] Gurung A Wrammert J Sunny AK Gurung R Rana N Basaula YN . Incidence, risk factors and consequences of preterm birth–findings from a multi-centric observational study for 14 months in Nepal. Arch Public Health. (2020) 78:1–9. 10.1186/s13690-020-00446-7 32695337 PMC7368758

[15] Hussein H Shamsipour M Yunesian M Hasanvand MS Fotouhi A. Association of adverse birth outcomes with exposure to fuel type use: a prospective cohort study in the northern region of Ghana. Heliyon. (2020) 6:6. 10.1016/j.heliyon.2020.e04169 32551393 PMC7287244

[16] Islam S Mohanty SK. Maternal exposure to cooking smoke and risk of low birth weight in India. Sci Total Environm. (2021) 774:145717. 10.1016/j.scitotenv.2021.145717 33609837

[17] Jiang M Qiu J Zhou M He X Cui H Lerro C . Exposure to cooking fuels and birth weight in Lanzhou, China: a birth cohort study. BMC Public Health. (2015) 15:1–10. 10.1186/s12889-015-2038-1 26215397 PMC4517486

[18] Flanagan E Oudin A Walles J Abera A Mattisson K Isaxon C . Ambient and indoor air pollution exposure and adverse birth outcomes in Adama, Ethiopia. Environ Int. (2022) 164:107251. 10.1016/j.envint.2022.107251 35533531

[19] Tapia VL Vasquez BV Vu B Liu Y Steenland K Gonzales GF. Association between maternal exposure to particulate matter (PM(2.5)) and adverse pregnancy outcomes in Lima, Peru. J Expo Sci Environ Epidemiol. (2020) 30:689–97. 10.1038/s41370-020-0223-5 32355212 PMC7853153

[20] Mulenga D. Maternal exposure to household air pollution and associated adverse birth outcomes in Ndola and Masaiti, Zambia. EC Pulmonol Respirat Med. (2018) 7:82–97.

[21] Suryadhi MAH Abudureyimu K Kashima S Yorifuji T. Effects of household air pollution from solid fuel use and environmental tobacco smoke on child health outcomes in Indonesia. J Occupat Environm Med. (2019) 61:335–9. 10.1097/JOM.0000000000001554 30724770

[22] Nisha MK Alam A Raynes-Greenow C. Variations in perinatal mortality associated with different polluting fuel types and kitchen location in Bangladesh. Int J Occup Environ Health. (2018) 24:47–54. 10.1080/10773525.2018.1507868 30156135 PMC6225514

[23] Bickton FM Ndeketa L Sibande GT Nkeramahame J Payesa C Milanzi EB. Household air pollution and under-five mortality in sub-Saharan Africa: an analysis of 14 demographic and health surveys. Environ Health Prev Med. (2020) 25:1–11. 10.1186/s12199-020-00902-4 33148165 PMC7643379

[24] Gebremeskel Kanno G Hussen Kabthymer R. Association of low birthweight with indoor air pollution from biomass fuel in sub-Saharan Africa: a systemic review and meta-analysis. Sustainable Environm. (2021) 7:1922185. 10.1080/27658511.2021.1922185

[25] Ghosh R Causey K Burkart K Wozniak S Cohen A Brauer M. Ambient and household PM_2.5_ pollution and adverse perinatal outcomes: a meta-regression and analysis of attributable global burden for 204 countries and territories. PLoS Med. (2021) 18:e1003718. 10.1371/journal.pmed.1003718 34582444 PMC8478226

[26] Page MJ McKenzie JE Bossuyt PM Boutron I Hoffmann TC Mulrow CD . The PRISMA 2020 statement: an updated guideline for reporting systematic reviews. Int J Surg. (2021) 88:105906. 10.1016/j.ijsu.2021.105906 33789826

[27] Joanna Briggs Institute. The Joanna Briggs Institute Critical Appraisal Tools for Use in JBI Systematic Reviews; Checklist for Case Control Studies. (2017). Available online at: http://joannabriggs.org/research/critical-appraisal-tools.html

[28] Moola S Munn Z Tufanaru C Aromataris E Sears K Sfetcu R . Chapter 7: Systematic reviews of etiology and risk. In: Joanna Briggs Institute Reviewer's Manual. Adelaide: The Joanna Briggs Institute. (2017) p. 5.

[29] Munn Z Moola S Lisy K Riitano D Tufanaru C. Methodological guidance for systematic reviews of observational epidemiological studies reporting prevalence and cumulative incidence data. JBI Evid Implement. (2015) 13:147–53. 10.1097/XEB.0000000000000054 26317388

[30] DerSimonian R Laird N. Meta-analysis in clinical trials revisited. Contemp Clin Trials. (2015) 45:139–45. 10.1016/j.cct.2015.09.002 26343745 PMC4639420

[31] Higgins JP Thompson SG. Quantifying heterogeneity in a meta-analysis. Stat Med. (2002) 21:1539–58. 10.1002/sim.1186 12111919

[32] Egger M SG Schneider M Minder C. Bias in meta-analysis detected by a simple, graphical test. BMJ. (1997) 315:629–34. 10.1136/bmj.315.7109.629 9310563 PMC2127453

[33] WHO. Low Birth Weight. Geneva: World Health Organization. (2024).

[34] WHO. World Health Organization: Stillbirth. (2023). Available online at: https://www.who.int/health-topics/stillbirth#tab=tab_1

[35] WHO. The Global Health Observatory: Neonatal Mortality Rate (0 to 27 days) per 1000 live births (SDG 3.2.2). (2024).

[36] WHO. World Health Organization: Preterm Birth. (2023). Available online at: https://www.who.int/news-room/fact-sheets/detail/preterm-birth#:~:text=Preterm%20is%20defined%20as%20babies,to%20less%20than%2032%20weeks

[37] Schlaudecker EP Munoz FM Bardají A Boghossian NS Khalil A Mousa H . Small for gestational age: case definition & guidelines for data collection, analysis, and presentation of maternal immunisation safety data. Vaccine. (2017) 35:6518. 10.1016/j.vaccine.2017.01.040 29150057 PMC5710996

[38] Ahmed Z Zafar M Khan NA Qureshi MS. Exposure to biomass fuel and low child birth weight–Findings of Pakistan Demographic and Health Survey 2006–2007. Int J Health Syst Disast Manage. (2015) 3:19. 10.4103/2347-9019.168569

[39] Bachwenkizi J Liu C Meng X Zhang L Wang W van Donkelaar A . Maternal exposure to fine particulate matter and preterm birth and low birth weight in Africa. Environm Int. (2022) 160:107053. 10.1016/j.envint.2021.107053 34942408

[40] Epuitai J Woolley KE Bartington SE Thomas GN. Association between wood and other biomass fuels and risk of low birthweight in Uganda: a cross-sectional analysis of 2016 Uganda demographic and health survey data. Int J Environ Res Public Health. (2022) 19:4377. 10.3390/ijerph19074377 35410058 PMC8999071

[41] Haider MR Rahman MM Islam F Khan MM. Association of low birthweight and indoor air pollution: biomass fuel use in Bangladesh. J Health Pollut. (2016) 6:18–25. 10.5696/2156-9614-6-11.18 30524794 PMC6221487

[42] Anil KC Basel PL Singh S. Low birth weight and its associated risk factors: health facility-based case-control study. PLoS ONE. (2020) 15:e0234907. 10.1371/journal.pone.0234907 32569281 PMC7307746

[43] Khan MN Nurs CZ Mofizul Islam M Islam MR Rahman MM. Household air pollution from cooking and risk of adverse health and birth outcomes in Bangladesh: a nationwide population-based study. Environm Health. (2017) 16:1–8. 10.1186/s12940-017-0272-y 28610581 PMC5470285

[44] Lakshmi P Virdi NK Sharma A Tripathy JP Smith KR Bates MN . Household air pollution and stillbirths in India: analysis of the DLHS-II National Survey. Environ Res. (2013) 121:17–22. 10.1016/j.envres.2012.12.004 23375552

[45] Li Q. Wang Y-y, Guo Y, Zhou H, Wang X, Wang Q, et al. Effect of airborne particulate matter of 25 μm or less on preterm birth: a national birth cohort study in China. Environm Int. (2018) 121:1128–36. 10.1016/j.envint.2018.10.025 30352698

[46] Mishra V Dai X Smith KR Mika L. Maternal exposure to biomass smoke and reduced birth weight in Zimbabwe. Ann Epidemiol. (2004) 14:740–7. 10.1016/j.annepidem.2004.01.009 15519895

[47] Mukherjee S Siddique S Chakraborty S Bhattacharya P Banerjee M Roychoudhury S . Adverse reproductive health outcomes in pre-menopausal Indian women chronically exposed to biomass smoke. J Public Health. (2015) 23:363–72. 10.1007/s10389-015-0690-7

[48] Pan D Liu S Huang D Zeng X Zhang Y Pang Q . Effects of household environmental exposure and ventilation in association with adverse birth outcomes: a prospective cohort study in rural China. Sci Total Environm. (2022) 822:153519. 10.1016/j.scitotenv.2022.153519 35101501

[49] Patel AB Meleth S Pasha O Goudar SS Esamai F Garces AL . Impact of exposure to cooking fuels on stillbirths, perinatal, very early and late neonatal mortality-a multicenter prospective cohort study in rural communities in India, Pakistan, Kenya, Zambia and Guatemala. Matern Health, Neonatol Perinatol. (2015) 1:1–12. 10.1186/s40748-015-0019-0 27057335 PMC4823690

[50] Roberman J Emeto TI Adegboye OA. Adverse birth outcomes due to exposure to household air pollution from unclean cooking fuel among women of reproductive age in Nigerias. Int J Environ Res Public Health. (2021) 18:634. 10.3390/ijerph18020634 33451100 PMC7828613

[51] Siddiqui AR Gold EB Yang X Lee K Brown KH Bhutta ZA. Prenatal exposure to wood fuel smoke and low birth weight. Environ Health Perspect. (2008) 116:543–9. 10.1289/ehp.10782 18414641 PMC2290983

[52] Sreeramareddy CT Shidhaye RR Sathiakumar N. Association between biomass fuel use and maternal report of child size at birth-an analysis of 2005-06 India Demographic Health Survey data. BMC Public Health. (2011) 11:1–10. 10.1186/1471-2458-11-403 21619613 PMC3125371

[53] Sunny S Mani B Mathew M D'Souza JM Jose A Johnson AR. Prenatal exposure to indoor air pollution and the risk of Low Birth Weight: a case-control study in a rural maternity hospital in Ramanagara district, Karnataka. Nat J Res Commun Med. (2019) 8:126–30. 10.26727/NJRCM.2019.8.2.126-130

[54] Tielsch JM Katz J Thulasiraj RD Coles CL Sheeladevi S Yanik EL . Exposure to indoor biomass fuel and tobacco smoke and risk of adverse reproductive outcomes, mortality, respiratory morbidity and growth among newborn infants in south India. Int J Epidemiol. (2009) 38:1351–63. 10.1093/ije/dyp286 19759098

[55] Vakalopoulos A Dharmage SC Dharmaratne S Jayasinghe P Lall O Ambrose I . Household air pollution from biomass fuel for cooking and adverse fetal growth outcomes in rural Sri Lanka. Int J Environ Res Public Health. (2021) 18:1878. 10.3390/ijerph18041878 33671963 PMC7918999

[56] Weber E Adu-Bonsaffoh K Vermeulen R Klipstein-Grobusch K Grobbee DE Browne JL . Household fuel use and adverse pregnancy outcomes in a Ghanaian cohort study. Reprod Health. (2020) 17:29. 10.1186/s12978-020-0878-3 32087720 PMC7036189

[57] Wylie BJ Coull BA Hamer DH Singh MP Jack D Yeboah-Antwi K . Impact of biomass fuels on pregnancy outcomes in central East India. Environm Health. (2014) 13:1. 10.1186/1476-069X-13-1 24405644 PMC3922846

[58] Xie G Sun L Yang W Wang R Shang L Yang L . Maternal exposure to PM_2.5_ was linked to elevated risk of stillbirth. Chemosphere. (2021) 283:131169. 10.1016/j.chemosphere.2021.131169 34146867

[59] Zhang H Zhang X Wang Q Xu Y Feng Y Yu Z . Ambient air pollution and stillbirth: an updated systematic review and meta-analysis of epidemiological studies. Environm Pollut. (2021) 278:116752. 10.1016/j.envpol.2021.116752 33689950

[60] Blencowe H Krasevec J De Onis M Black RE An X Stevens GA . National, regional, and worldwide estimates of low birthweight in 2015, with trends from 2000: a systematic analysis. Lancet Global Health. (2019) 7:e849–e60. 10.1016/S2214-109X(18)30565-5 31103470 PMC6560046

[61] Kadam YR Mimansa A Chavan PV Gore AD. Effect of prenatal exposure to kitchen fuel on birth weight. Indian J Community Med. (2013) 38:212. 10.4103/0970-0218.120155 24302821 PMC3831690

[62] Oliveira BFAd Ignotti E Hacon SS. A systematic review of the physical and chemical characteristics of pollutants from biomass burning and combustion of fossil fuels and health effects in Brazil. Cadernos de saude publica. (2011) 27:1678–98. 10.1590/S0102-311X2011000900003 21986597

[63] Woolley KE Dickinson-Craig E Bartington SE Oludotun T Kirenga B Mariga ST . Effectiveness of interventions to reduce household air pollution from solid biomass fuels and improve maternal and child health outcomes in low-and middle-income countries: a systematic review protocol. Syst Rev. (2021) 10:1–7. 10.1186/s13643-021-01590-z 33472668 PMC7818907

[64] DeFranco E Moravec W Xu F Hall E Hossain M Haynes EN . Exposure to airborne particulate matter during pregnancy is associated with preterm birth: a population-based cohort study. Environm Health. (2016) 15:1–8. 10.1186/s12940-016-0094-3 26768419 PMC4714531

[65] Zhu X Liu Y Chen Y Yao C Che Z Cao J. Maternal exposure to fine particulate matter (PM_2.5_) and pregnancy outcomes: a meta-analysis. Environm Sci Pollut Res. (2015) 22:3383–96. 10.1007/s11356-014-3458-7 25163563

[66] Naz S Page A Agho KE. Household air pollution from use of cooking fuel and under-five mortality: the role of breastfeeding status and kitchen location in Pakistan. PLoS ONE. (2017) 12:e0173256. 10.1371/journal.pone.0173256 28278260 PMC5344381

[67] Kleimola LB Patel AB Borkar JA Hibberd PL. Consequences of household air pollution on child survival: evidence from demographic and health surveys in 47 countries. Int J Occup Environ Health. (2015) 21:294–302. 10.1179/2049396715Y.0000000007 25843087 PMC4727588

[68] Liu S Krewski D Shi Y Chen Y Burnett RT. Association between gaseous ambient air pollutants and adverse pregnancy outcomes in Vancouver, Canada. Environ Health Perspect. (2003) 111:1773–8. 10.1289/ehp.6251 14594630 PMC1241722

[69] Ritz B Yu F Chapa G Fruin S. Effect of air pollution on preterm birth among children born in Southern California between 1989 and 1993. Epidemiology. (2000) 11:502–11. 10.1097/00001648-200009000-00004 10955401

[70] Sagiv SK Mendola P Loomis D Herring AH Neas LM Savitz DA . A time series analysis of air pollution and preterm birth in Pennsylvania, 1997–2001. Environ Health Perspect. (2005) 113:602–6. 10.1289/ehp.7646 15866770 PMC1257554

[71] Anwar A Ayub M Khan N Flahault A. Nexus between air pollution and neonatal deaths: a case of Asian countries. Int J Environ Res Public Health. (2019) 16:4148. 10.3390/ijerph16214148 31661852 PMC6861973

