# Supplementary material for: Exposure to indoor air pollution and adverse pregnancy outcomes in low and middle-income countries: a systematic review and meta-analysis
Source: Front Public Health. 2024 May 22;12:1356830. doi: 10.3389/fpubh.2024.1356830 (PMC11151685; doi:10.3389/fpubh.2024.1356830)
Supplement: Supplementary file 2 [file Table_2.DOCX]

Figure 1: Sensitivity analysis of pooled association between indoor air pollution exposure and low birth weight in low and middle-income countries, 2023

Figure 2: Sensitivity analysis of pooled association between indoor air pollution exposure and small for gestational age in low and middle-income countries, 2023

Figure 3: Sensitivity analysis of pooled association between indoor air pollution exposure and preterm birth in low and middle-income countries, 2023

Figure 4: Sensitivity analysis of pooled association between indoor air pollution exposure and neonatal death in low and middle-income countries, 2023
